# Erythrocyte tropism of malarial parasites: The reticulocyte appeal

**DOI:** 10.3389/fmicb.2022.1022828

**Published:** 2022-10-26

**Authors:** Yew Wai Leong, Bruce Russell, Benoit Malleret, Laurent Rénia

**Affiliations:** ^1^A*STAR Infectious Diseases Labs, Agency for Science, Technology and Research, Singapore, Singapore; ^2^Department of Microbiology and Immunology, University of Otago, Dunedin, New Zealand; ^3^Singapore Immunology Network, Agency for Science, Technology and Research, Singapore, Singapore; ^4^Department of Microbiology and Immunology, Immunology Translational Research Program, Yong Loo Lin School of Medicine, National University of Singapore, Singapore, Singapore; ^5^Lee Kong Chian School of Medicine, Nanyang Technological University, Singapore, Singapore; ^6^School of Biological Sciences, Nanyang Technological University, Singapore, Singapore

**Keywords:** reticulocyte, normocyte, erythrocyte tropism, erythrocyte invasion, bone marrow, gametocyte, zoonotic malaria

## Abstract

Erythrocytes are formed from the enucleation of erythroblasts in the bone marrow, and as erythrocytes develop from immature reticulocytes into mature normocytes, they undergo extensive cellular changes through their passage in the blood. During the blood stage of the malarial parasite life cycle, the parasite sense and invade susceptible erythrocytes. However, different parasite species display varying erythrocyte tropisms (i.e., preference for either reticulocytes or normocytes). In this review, we explore the erythrocyte tropism of malarial parasites, especially their predilection to invade reticulocytes, as shown from recent studies. We also discuss possible mechanisms mediating erythrocyte tropism and the implications of specific tropisms to disease pathophysiology. Understanding these allows better insight into the role of reticulocytes in malaria and provides opportunities for targeted interventions.

## Introduction

Mature erythrocytes are biconcave enucleated cells containing hemoglobin, which transport oxygen and carbon dioxide between the lungs and tissues. As with all blood cells, erythrocytes are formed from hematopoietic stem cells (HSCs) primarily in the adult bone marrow, in a process known as erythropoiesis. Classically, early erythropoiesis is thought to occur in a hierarchical fashion, with HSCs forming common myeloid progenitors (CMPs), then megakaryocyte-erythroid progenitors (MEPs), before bifurcating and committing into the megakaryocyte and erythroid lineages. However, recent studies have challenged this classical model and proposed alternative differentiation pathways leading up to the erythroid lineage (Dulmovits et al., 2017; Xavier-Ferrucio and Krause, 2018), including the model of heterogenous CMPs comprising unipotent subpopulations with either myeloid, erythroid or megakaryocyte potential (Perié et al., 2015; Notta et al., 2016). The earliest progenitor committed to the erythroid lineage is the burst-forming unit-erythroid (BFU-E) cells, which then differentiates into the colony-forming unit-erythroid (CFU-E) cells (Stephenson et al., 1971; Gregory et al., 1973; Gregory and Eaves, 1977, 1978). Terminal erythropoiesis begins once proerythroblasts (the earliest morphologically recognizable erythroid) are formed from CFU-E cells. The proerythroblast then undergoes multiple rounds of mitosis (Hu et al., 2013; Liu et al., 2013) to sequentially form the basophilic, polychromatic and orthochromatic erythroblasts. While it has been shown that late-stage erythroblasts can indeed be invaded by malaria parasites (Panichakul et al., 2007; Tamez et al., 2009), there is little evidence to suggest that robust asexual intraerythrocytic parasite development occur in erythroblasts. It is only after the orthochromatic erythroblast eject its nucleus to form the nascent reticulocyte that they become fully amenable to *Plasmodium* spp. invasion and replication.

In this review, we focus on the role of reticulocytes in malaria pathogenesis, but before that, it is important to provide some background to the neglected field of reticulocyte biology. After enucleation, it takes 2–3 days for reticulocytes to fully mature into normocytes (Baldini and Pannacciulli, 1960; Gronowicz and Swift, 1984; Koury et al., 2005). Throughout this period, reticulocytes undergo extensive cellular changes including the transformation from large, complex and rigid early reticulocytes into small, simple and deformable normocytes (Heilmeyer and Westhäuser, 1932; Malleret et al., 2013). Reticulocyte transformation is achieved by expelling large volumes of their membrane and cytoplasm *via* vesicle shedding, and by controlling water content *via* transporters (Quarmyne et al., 2011; Griffiths et al., 2012; Gallagher, 2017; Ovchynnikova et al., 2018). Interestingly, reticulocyte maturation (vesicle shedding in particular) was suggested to be driven by shear stress in the circulation (Moura et al., 2018, 2020). Reticulocytes complete the synthesis of hemoglobin during this maturation period, although the majority of hemoglobin were already synthesized during erythroblast development (Skadberg et al., 2003). Residual organelles like mitochondria and ribosomes are also degraded and expelled from the cell (Gronowicz and Swift, 1984; Koury et al., 2005; Moras et al., 2017). More drastically, the reticulocyte membrane and cytoskeleton undergo significant remodeling (Ovchynnikova et al., 2018), resulting in the uniformly biconcave erythrocyte, with highly deformable properties to traverse tiny capillaries and splenic interendothelial slits (Buffet et al., 2006, 2009). Reticulocyte membrane remodeling also affects its surface proteome, during which many surface proteins are lost (Malleret et al., 2013; Wilson et al., 2016; Chu et al., 2018). Most notably, CD71 (transferrin receptor 1) which is highly abundant on erythroblasts and early reticulocytes (to import iron for hemoglobin synthesis), drops to undetectable levels on normocytes. During reticulocyte maturation, instead of being recycled back to the surface post-internalization, CD71 is rerouted to the exosomal pathway and expelled (Pan and Johnstone, 1983, 1984; Pan et al., 1985; Johnstone et al., 1987). A closer look at the exosomes released by reticulocytes will reveal the presence of other membrane proteins, such as α4β1 integrin (Rieu et al., 2000), aquaporin-1 (Blanc et al., 2009), CD55 (Rabesandratana et al., 1998) and CD59 (Rabesandratana et al., 1998), indicating that the exosomal pathway is the main mechanism of protein removal in reticulocytes. Altogether, these multifaceted changes during reticulocyte maturation lead to the formation of normocytes with a drastically different phenotype.

The blood stage of malaria involves the invasion of host erythrocytes by *Plasmodium* parasites. Infective merozoites sense and bind to receptors on the erythrocyte surface, initiating the complex invasion process and resulting in parasite internalization (Weiss et al., 2015; Cowman et al., 2017). Within erythrocytes, developing parasites mainly acquire nutrients from the host erythrocytes (especially hemoglobin), but the parasites also import additional nutrients from the extracellular milieu (Counihan et al., 2021). To this end, the parasites remodel their host cells by exporting hundreds of *Plasmodium* proteins to the erythrocyte membrane and cytosol (de Koning-Ward et al., 2016). This extensive remodeling, besides promoting nutrient uptake, also enables the infected cells to escape splenic and immune clearance.

The *Plasmodium* parasite, however, does not just invade any erythrocytes; erythrocytes from certain blood groups and disorders show increased resistance to merozoite invasion and parasite development (Langhi and Orlando Bordin, 2006; Rowe et al., 2009; Taylor et al., 2013). From the known phenotypic differences between these erythrocyte types, we understand to a certain degree, how the differential invasion capacity of malarial parasites into these erythrocyte types shaped human evolution (Elguero et al., 2015). On the contrary, although we are starting to appreciate the stark dissimilarities between reticulocytes and normocytes, not much is known regarding the implications of reticulocyte and normocyte tropisms. Here, we first summarize tropism studies to better understand the erythrocyte preferences of relevant malaria parasites. Realizing that most *Plasmodium* spp. show a predilection for reticulocytes, we then discuss potential mechanisms on how the parasites seek and invade host reticulocytes. Finally, we explore possible reasons for reticulocyte tropism, and the implications of this to malaria outcome and intervention strategies.

## Erythrocyte tropism of human, non-human primate, and rodent malaria

### Reticulocyte characterization

Due to extensive cellular remodeling during reticulocyte maturation, the reticulocyte population is highly heterogenous and strikingly different from the final normocyte stage. Human reticulocytes in circulation were first categorized into four groups (using brilliant Cresyl blue staining on blood smears) by Heilmeyer in 1932 (Heilmeyer and Westhäuser, 1932). New methylene blue soon replaced brilliant Cresyl blue as the standard reticulocyte stain, and is still used to this day, due to its clear deep blue staining of reticular matter, enabling easy reticulocyte quantification (Brecher, 1949). Alternatively, the Giemsa-stained wet mount method was also developed for reticulocyte quantification (Lee et al., 2013). As cellular phenotyping progressed from traditional light microscopy to flow cytometry, thiazole orange (TO) became widely used as a marker of reticulocyte maturity, for the reason that its excitation and emission profiles are detectable by most flow cytometers (Lee et al., 1986; Davis et al., 1995). However, erythrocytic parasites and inclusions, such as *Plasmodium* spp. and Howell-Jolly bodies, could give false positives with TO staining. Measuring CD71 loss as reticulocytes mature is more specific, and better defined characterization of reticulocyte stages were achieved when both TO and CD71 are used together (Bhardwaj and Saxena, 2013; Malleret et al., 2013; Ovchynnikova et al., 2017). For malaria studies, however, CD71 may not be an accurate marker for reticulocyte maturity as *P. vivax*-infected reticulocytes rapidly lose CD71 post-invasion (Malleret et al., 2015). Utilizing additional reticulocyte markers like CD98, alongside CD71 and TO, should circumvent such problems (Griffiths et al., 2012; Leong et al., 2021; Malleret et al., 2021).

It is worth noting, however, that some *in vivo* studies did detect reticular matter and CD71 on *P. vivax*-infected reticulocytes (Lim et al., 2016; Clark et al., 2021). Certainly, Malleret et al. also observed some CD71-positive *P. vivax*-infected reticulocytes taken from 13 vivax malaria patients, however, these cells only comprised 1.29% of the total infected cell population (Malleret et al., 2015). If CD71 is indeed important for *P. vivax* invasion of reticulocytes, it would presumably be an important fitness advantage for the parasite to rapidly eject these receptors (i.e., CD71), to restrict further invasion by additional merozoites into the same cell (Malleret et al., 2017). While multiple *P. vivax* invasions of reticulocytes are indeed common, in almost all cases observed (hundreds of *in vivo* patient isolates and *ex vivo* assays conducted by the authors; unpublished observations), these have occurred simultaneously (the developmental stages of the parasites within the same erythrocytes were synchronous), and we have yet to observe reinvasion of an erythrocyte already containing a mature stage parasite.

### Tropism studies

Considering the heterogeneity of erythrocytes, it is unsurprising that different *Plasmodium* spp. display varying preferences for host erythrocyte types. Erythrocyte tropism is a key feature of *Plasmodium* spp. pathogenesis and many studies have attempted to characterize merozoite erythrocyte preference. These tropism studies are summarized in Table 1 for relevant parasite species infecting humans (*P. falciparum*, *P. vivax*, *P. malariae*, *P. ovale, P. knowlesi*, and *P. cynomolgi*) and rodents (*P. berghei*, *P. yoelii*, *P. chabaudi*, and *P. vinckei*). Although *P. knowlesi* and *P. cynomolgi* infections are not formally classified as human malaria, zoonotic transmissions to humans are emerging (Singh et al., 2004; Ta et al., 2014; Imwong et al., 2019) and their tropism for human erythrocytes have been studied, hence their inclusion in this review.

**Table 1:** Erythrocyte tropism of human- and rodent-infecting *Plasmodium* species.

Parasite species	Parasite strain/isolate	Host erythrocyte	Technique	Tropism	Details	References
*P. falciparum*	Patient isolate	Human	PB smear	Mixed	“Infected RET to Infected NORM” ratio comparable to “Total RET to Total NORM” ratio, suggesting no preference	Shushan et al. (1937)
	Patient isolate	Human	PB smear	RET	RET parasitemia 14X higher than NORM parasitemia	Hegner (1938)
	Patient isolate	Human	PB smear	Mixed	NORM parasitemia was higher in some observations and RET parasitemia was higher in others	Kitchen (1939)
	Patient isolate	Human	Invasion assay and smear	RET	Higher invasion rates (2.4–2.8X) with the younger RBC fraction	Pasvol et al. (1977)
	Patient isolate	Human	PB smear; Invasion assay and smear	RET	RET parasitemia 1.6–4.1X higher than NORM parasitemia. Invasion rates in younger RBC fraction were 2.0–4.5X higher than older RBC fraction	Pasvol et al. (1980)
	3D7 clone	Human	Invasion assay and smear	RET	Higher invasion rates with the younger RBC fractions	Lim et al. (2013)
	3D7 clone	Human	Invasion assay and smear	RET	Invasion into CD71+ RBCs was 2X higher than CD71- RBCs. Invasion rates also positively correlated with % of CD71+ RBCs	Naidu et al. (2019)
*P. vivax*	Patient isolate	Human	PB smear	RET	RET parasitemia 1292X higher than NORM parasitemia	Hegner (1938)
	Patient isolate	Human	PB smear	RET	RET parasitemia consistently higher than NORM parasitemia throughout infection	Kitchen (1938)
	Patient isolate	Human	PB smear; Invasion assay and smear	RET	RET parasitemia 180X and 52X higher than NORM parasitemia *in vivo* and *in vitro*, respectively	Mons et al. (1988)
	Thai patient isolate	Human	Invasion assay and flow cytometry	RET	Invasion rates into CD71+ RBCs were the highest	Malleret et al. (2015)
	Indian patient isolate	Human	PB smear	RET	Highly variable RET preferences across different parasite isolates: RET parasitemia 11-519X higher than NORM parasitemia	Lim et al. (2016)
*P. malariae*	Patient isolate	Human	PB smear	RET	RET parasitemia 10X higher than NORM parasitemia	Hegner (1938)
	Patient isolate	Human	PB smear	NORM	Infected RETs were only found in 17% of observations	Kitchen (1939)
	Patient isolate	Human	PB smear	NORM	NORM parasitemia 2.9X higher than RET parasitemia	Chwatt (1948)
*P. ovale*	Patient isolate	Human	PB smear	RET	Half of young trophozoites were found in RETs, but the true proportion of RET at the point of invasion was likely higher	Garnham et al. (1955)
*P. knowlesi*	H strain: WT and human-adapted	Human	Invasion assay and smear	WT: RET; Human-adapted: Mixed	WT strain had higher invasion into younger RBC fractions. Human-adapted strain invaded both young and old RBC fractions similarly	Lim et al. (2013)
	A1-H.1 clone; UM01 strain	Human; Cynomolgus macaque	Invasion assay and smear	Human RET; Mixed tropism for macaque RBCs	Both parasite lines invaded human RETs more efficiently than human NORMs. Both lines invaded macaque RETs and NORMs similarly	Amir et al. (2016)
	A1-O strain; A1-C.1 clone (cynomolgus-adapted); A1-H.1 clone (human-adapted)	Human	Invasion assay and smear	Human RET	All parasite lines showed a slight increase in invasion into RET-enriched human RBCs compared to normal human RBCs	Moon et al. (2016)
*P. cynomolgi*	B strain	Human; Rhesus macaque	Invasion assay and smear	Human RET; Mixed tropism for macaque RBCs	Only invaded CD71+ CD234+ human RETs but not NORMs. Invaded macaque RBCs indiscriminately	Kosaisavee et al. (2017)
*P. berghei*	KBG 173 strain	CF1	PB smear	RET	At equal RET and NORM frequencies, 41% of RETs were infected but only 1% of NORMs were infected	Singer (1954)
	K173 strain	B10.LP	PB smear	RET	In phlebotomized mice, >60% of parasites were within RETs, as compared to mice that were not bled, most parasites were in NORMs	Schetters et al. (1986)
	ANKA strain	BALB/c; Schofield; CD-1	Invasion assay and smear	RET	Invasion rates into RBCs of younger mice and mice treated with PHZ were much higher, presumably due to the higher RET proportion	McNally et al. (1992)
	ANKA strain; NK65 strain	Swiss albino	PB smear	ANKA: NORM; NK65: RET	ANKA: 80% of parasites were found in NORMs; NK65: 50% of parasites were found in NORMs, but after PHZ treatment, 85% of parasites were within RETs	Deharo et al. (1996)
	ANKA strain	BALB/c	PB smear	RET	The % of RETs that were infected were higher than NORM throughout infection	Cromer et al. (2006)
	ANKA strain	C57BL/6	Flow cytometry on PB	RET	Used CD71 as a RET marker. Observed that RETs were invaded 1.46 times more frequently than NORMs	Thakre et al. (2018)
	ANKA strain (clone 15cy1)	C57BL/6	Invasion assay and flow cytometry	RET	Prefers CD71+ CD98+ RETs 5X more than CD71- CD98- NORMs	Leong et al. (2021)
*P. yoelii yoelii*	17XL strain (clone YM); A/C strain	C57 black	PB smear	17XL: NORM; A/C: RET	N.R.	Walliker et al. (1976)
	17XNL strain; 17XL strain (clone YM)	A/Tru	PB smear	RET	Exclusively in RET of PHZ-treated mice	Fahey and Spitalny (1984)
	265BY strain	C57BL/6	PB smear	RET	Most of infected cells (60%–100%) were RET	Vigário et al. (2001)
	17XNL strain; 17XL strain	BALB/c	PB smear	17XNL: RET; 17XL: NORM	17XNL: >90% RET were infected; 17XL: <1% RET were infected	Shi et al. (2005)
	17XNL strain; 17XL strain	BALB/c	PB smear	17XNL: RET; 17XL: Mixed	Erythrocyte preference was determined using selectivity index, a measure based on multiply infected erythrocytes	Otsuki et al. (2009)
	17XNL strain	BALB/c	Flow cytometry on PB	RET	Prefers to invade CD71-high RET	Martín-Jaular et al. (2013)
	17XNL strain	C57BL/6	Flow cytometry on PB	RET	*In vivo* biotinylation to differentiate young and aged RBCs. Higher % of young RBCs invaded than aged RBCs	Banerjee et al. (2015)
	17XNL strain	C57BL/6	Flow cytometry on PB	RET	Used CD71 as a RET marker. Higher % of RET invaded compared to NORM	Hojo-Souza et al. (2020)
	17XNL strain (clone 1.1)	C57BL/6	Invasion assay and flow cytometry	RET	Prefers CD71+ CD98+ RETs 2.5X more than CD71- CD98- NORMs	Leong et al. (2021)
*P. chabaudi chabaudi*	N.R.	CF1	PB smear	Mixed	The % of RET and NORM that were infected were similar throughout infection in PHZ mice. % of RET invaded was lower than that for *P. berghei* (around 1/3 of the value)	Ott (1968)
	Clone AS	CBA/Ca	PB smear	Mixed (slight NORM preference)	When RET and NORM were at equal frequencies, infected NORM comprised 64% of total infected RBCs	Jarra and Brown (1989)
	Clone AS	BALB/c; Schofield; CD-1	Invasion assay and smear	Mixed (slight RET preference)	Invasion rates into RBCs of younger mice and mice treated with PHZ were slightly higher, presumably due to the higher RET proportion. RET preference not as high as *P. berghei*	McNally et al. (1992)
	Clone AS	C57BL/6	Invasion assay and flow cytometry	Mixed	Indiscriminately invaded CD71+ CD98+ RETs and CD71- CD98- NORMs	Leong et al. (2021)
*P. vinckei*	N.R.	CF1	PB smear	NORM	In PHZ mice at peak parasitemia, 98% of NORMs were infected but only 35% of RETs were infected.	Viens et al. (1971)
	106HW strain	C57BL/6	PB smear	NORM	100% of infected cells were NORMs	Vigário et al. (2001)
	S67 strain	C57BL/6	Invasion assay and flow cytometry	Mixed	Indiscriminately invaded CD71+ CD98+ RETs and CD71- CD98- NORMs	Leong et al. (2021)

RET, Reticulocyte; NORM, Normocyte; RBC, Red blood cell; PB, Peripheral blood; PHZ, Phenylhydrazine; N.R., Not reported.

The most common method employed to study erythrocyte tropism is microscopic evaluation of peripheral blood smears taken from infected patients and animals, followed by comparing the *in vivo* parasite distribution in reticulocytes and normocytes. Due to the different frequencies of reticulocytes and normocytes in circulation, reticulocyte and normocyte parasitemia (% of reticulocytes and normocytes infected, respectively) are instead compared to deduce tropism. Observations of peripheral blood smears have limitations (Box 1), therefore some studies performed *ex vivo* invasion assays, comparing invasion into reticulocyte- and normocyte-enriched fractions. Besides microscopy, flow cytometry has also been used to enumerate infected cells and classify erythrocyte types (Malleret et al., 2011).

BOX 1. **Possible limitations of tropism studies.**

- Microscopic evaluations of infected reticulocytes vs. infected normocytes are subjective. Late-stage parasites also usually occupy most of the erythrocyte cytoplasm, possibly obscuring the reticular matter necessary for reticulocyte identification.
- Flow cytometric tropism studies typically use CD71 as a reticulocyte marker, which could be rapidly lost from infected reticulocytes, as seen with *P. vivax*.
- Human reticulocytes are commonly enriched by magnetic-activated cell sorting (MACS) using anti-CD71 antibodies conjugated to magnetic beads. As *P. vivax* use CD71 as an entry receptor, presence of anti-CD71 antibodies likely interferes with invasion measurements of *P. vivax*.
- Peripheral blood data are only a single-timepoint ‘snapshot’ of erythrocyte types during sample collection and do not represent the erythrocyte stage at the point of merozoite invasion.
- Infected reticulocytes and infected normocytes have different susceptibilities to the host immune system, resulting in different clearance rates *in vivo*.
- Parasite sequestration and extravascular parasite populations mean that peripheral blood data is not indicative of the whole parasite population in the host.

Most tropism studies on *P. falciparum* agree that it has the capability to invade both reticulocytes and normocytes but prefers reticulocytes when available (Table 1). Varying extents of its reticulocyte preference were reported: from as low as 2 times more (Pasvol et al., 1980; Naidu et al., 2019), to as high as 14 times more (Hegner, 1938) than its normocyte preference. While *P. falciparum* has a predilection for reticulocytes, *P. vivax* invasion is strictly restricted to reticulocytes, with an early report showing a 1,291-fold preference for reticulocytes over normocytes (Hegner, 1938). In fact, *P. vivax*’s exclusivity is amplified by observations that it only invades nascent reticulocytes, which express CD71 and CD98, both of which are *P. vivax*’s invasion receptors (Malleret et al., 2015, 2021; Gruszczyk et al., 2018). Contrastingly, a recent study involving a large Indian cohort of *P. vivax* isolates revealed substantial variation in the extent of reticulocyte preference (11- to 519-folds) across different isolates (Lim et al., 2016). This, however, could be confounded by the accelerated maturation of *P. vivax*-infected reticulocytes, resulting in the misidentification of infected cells as normocytes (Malleret et al., 2015). A follow-up study also using Indian *P. vivax* isolates showed large variations in the parasite’s dependency on the DARC (Duffy antigen receptor for chemokines) and CD71 receptors, possibly explaining its varying reticulocyte tropism reported earlier (Kanjee et al., 2021). On the other end, it could be worthwhile to compare the extent of reticulocyte preference of different Brazilian and Thai *P. vivax* isolates [which were used in Gruszczyk et al. (2018) and Malleret et al. (2021)], considering that they were shown to invade only a small subset of reticulocytes. Better characterization of *P. vivax* invasion into different reticulocyte subsets should also help to understand *P. vivax* invasion in Duffy-negative patients, particularly in Africa (Dechavanne et al., 2018; Baird, 2022).

As for *P. malariae*, studies on its erythrocyte tropism are limited and contradictory, with both reticulocyte tropism (Hegner, 1938) and normocyte tropism (Kitchen, 1939; Chwatt, 1948) observed. *P. ovale* is commonly cited to prefer reticulocytes (Kerlin and Gatton, 2013; Neveu and Lavazec, 2021), but primary evidence is limited (Garnham et al., 1955). Interestingly, the zoonotic *P. knowlesi* and *P. cynomolgi* have no invasion bias with macaque erythrocytes, but displayed a preference for human reticulocytes over human normocytes (Amir et al., 2016; Kosaisavee et al., 2017). In fact, the *P. cynomolgi* B strain showed negligible invasion into human normocytes, whereas its human reticulocyte invasion was comparable to its normal invasion into macaque erythrocytes (Kosaisavee et al., 2017). This has interesting implications for the zoonotic potential of NHP malaria strains (discussed further in Section “Zoonotic potential”).

Tropism studies on rodent malaria strains are more abundant due to the long history of malaria research on rodent models (Table 1). Despite the ease of manipulation of rodent models, most of these studies just looked at peripheral blood samples (which could be potentially skewed by *in vivo* confounders; Box 1) of infected animals to quantify erythrocyte tropism. Additionally, many neglected to objectively quantify the extent of reticulocyte or normocyte preference. Realizing this, we recently designed an *ex vivo* flow cytometric tropism assay and compared the erythrocyte tropism of multiple rodent malaria strains (Leong et al., 2021). We showed in a controlled environment where reticulocytes and normocytes are equally abundant, *P. berghei* (ANKA strain, clone 15cy1) displayed the strongest reticulocyte preference, with reticulocyte invasion 5 times higher than normocyte invasion. Besides that, *P. yoelii* (17X strain, clone 1.1) preferred reticulocytes 2.5 times more than normocytes, whereas *P. chabaudi* (clone AS) and *P. vinckei* (S67 strain) showed no particular tropisms. We hope that by ranking these different strains based on their erythrocyte tropism, future work can properly utilize these rodent malaria strains to model various human malaria invasions.

## Mechanisms mediating reticulocyte tropism

An interesting recurrence among most *Plasmodium* species mentioned above is reticulocyte tropism. Contrastingly, the other end of the spectrum (i.e., normocyte preference or exclusivity) is rarely reported, which is surprising considering that normocytes constitute the bulk of circulating erythrocytes and therefore, readily accessible by the parasite. The obvious question to ask is how do merozoites sense and recognize this rare reticulocyte population in an environment where normocytes are abundant? The prevalent hypothesis is that the recognition of reticulocyte-specific receptors confers the parasites their reticulocyte preference. This is exemplified by *P. vivax*’s recognition of human CD71 and CD98, by reticulocyte binding protein 2b and 2a (PvRBP2b and PvRBP2a), respectively (Gruszczyk et al., 2018; Malleret et al., 2021). It was also suggested recently that the reticulocyte-preferring *P. berghei* ANKA uses murine CD71 as an entry receptor (Hentzschel et al., 2022). Other *P. vivax* ligands like erythrocyte binding protein 2 (PvEBP2) and PvRBP1a were also shown to preferentially bind to human reticulocytes (Ntumngia et al., 2016, 2018), suggesting their possible interaction with CD71, CD98 or unidentified reticulocyte-specific receptors. However, utilization of reticulocyte-specific receptors does not explain the tropism signature of most parasite strains, which is preferring reticulocytes yet maintaining their normocyte invasive capabilities, with *P. falciparum* being the most notable example. In fact, *P. falciparum*’s ability to invade erythrocyte of various ages is commonly cited as the reason for its higher virulence compared to *P. vivax*. The elevated expression of the essential *P. falciparum* receptor, basigin (CD147), on early reticulocytes (Griffiths et al., 2012; Malleret et al., 2013) could augment merozoite binding and invasion into reticulocytes, but this has not been directly shown. CD147 detection by mass cytometry, however, did not reveal any changes in expression during reticulocyte maturation (Thomson-Luque et al., 2018).

Besides receptor abundance, another possible mechanism behind reticulocyte tropism is receptor remodeling during reticulocyte maturation. Ovchynnikova et al. showed that although DARC expression levels remained constant throughout reticulocyte maturation, there were increased binding of certain monoclonal anti-DARC antibodies and recombinant *P. vivax* Duffy binding protein (PvDBP) to early CD71-high reticulocytes, as compared to older reticulocyte subsets and normocytes (Ovchynnikova et al., 2017). These data imply that the DBP binding site on erythrocytic DARC is remodeled during reticulocyte maturation, abolishing DBP binding to mature erythrocytes and consequently, resulting in *P. vivax*’s restriction to early reticulocytes. However, it is unclear whether the change in DARC conformation directly impacts *P. vivax* merozoite invasion. Indeed, a recent study strongly suggested that the DARC-PvDBP interaction does not mediate reticulocyte specificity of *P. vivax* (Mohring et al., 2019). Since *P. knowlesi* also depends on human DARC for cell entry (Haynes et al., 1988; Moon et al., 2013), Mohring et al. generated transgenic *P. knowlesi* expressing PvDBP and found that its invasion into human normocytes was comparable to wildtype *P. knowlesi* expressing PkDBPα, suggesting that PvDBP does not mediate reticulocyte restriction. Nonetheless, further research is needed to reconcile this discrepancy and to properly delineate the contributions of DARC and PvDBP to *P. vivax*’s reticulocyte exclusivity.

Other than receptor-ligand interactions, the biophysical differences between reticulocytes and normocytes, especially cell deformability, could play a role in determining tropism. Merozoite binding to erythrocyte surface results in a strong deformation event prior to merozoite internalization, and video microscopy evidence showed that successful merozoite invasions correlated strongly with deformation strength of the erythrocyte membrane during this period (Weiss et al., 2015). Membrane deformability depends on membrane tension (surface stress) and viscoelasticity (membrane stiffness). In the context of malaria, membrane viscoelasticity is more commonly used as a metric of deformability, as measured by methods like micropipette aspiration, ektacytometry and atomic force microscopy (Russell and Cooke, 2017; Depond et al., 2020; Groomes et al., 2022). Membrane viscoelasticity increases during reticulocyte maturation resulting in the highly deformable normocyte (Chasis et al., 1989; Malleret et al., 2013). Although there is no direct evidence showing that membrane viscoelasticity affects invasion, it was suggested that uninfected erythrocytes are remodeled to be less stiff to facilitate future merozoite invasion (Dekel et al., 2021). Conversely, a direct link between erythrocyte membrane tension and merozoite invasion was reported recently; above a certain membrane tension threshold, merozoite invasion fails to occur (Kariuki et al., 2020). In this study, Kariuki et al. showed that erythrocytes from the Dantu blood group had higher membrane tensions than non-Dantu erythrocytes, and this feature provided significant protection against *P. falciparum* invasion. They also discussed the possibility that the lower membrane tension of reticulocytes (due to their larger size) compared to normocytes could explain *P. falciparum*’s preference for reticulocytes. Overall, although there are marked differences in reticulocyte and normocyte deformability under normal conditions, it is unclear for now whether reticulocytes and normocytes deform differently during the deformation stage of invasion in response to merozoite binding.

Earlier we asked the question of how do merozoites sense the scarcely available reticulocytes in the normocyte-rich circulation. Perhaps this is irrelevant as there is growing evidence that many *Plasmodium* species are able to invade and replicate within extravascular hematopoietic sites like the bone marrow and spleen (Imai et al., 2013; Joice et al., 2014; De Niz et al., 2018; Lee et al., 2018; Obaldia et al., 2018; Venugopal et al., 2020; Kho et al., 2021). At these reticulocyte-rich sites, reticulocyte invasion would be the norm instead of the exception. In light of this, although reticulocyte invasion studies had mostly focused on *P. vivax*, reticulocyte invasion for *P. falciparum* could be more common than we once thought. There is an urgent need to better understand this considering that reticulocyte tropism has important implications, as we shall discuss in the next section.

## Advantages, disadvantages, and implications of erythrocyte tropism

The ubiquity of reticulocyte tropism among *Plasmodium* spp. implies an inherent advantage to invading and developing within reticulocytes. At first glance, the most obvious advantage is that the younger age of reticulocytes confers longer survival in circulation. However, the parasite’s relatively short 24–72 h intraerythrocytic cycle as compared to human erythrocytes’ 120 days lifespan means that there is usually an abundance of circulating normocytes that can last long enough to sustain the parasite’s intraerythrocytic cycle. In this section, we explore other possible reasons for reticulocyte tropism.

### Immune implications

Although CD8+ T cell-mediated immunity is vital during liver stage malaria, CD8+ T cells were previously thought to play little to no protective role during blood stage malaria (Kurup et al., 2019), due to the lack of major histocompatibility complex class I (MHC-I) expression on erythrocytes (Kumar and Miller, 1990; Vinetz et al., 1990). However, there were some indications that reticulocytes still express MHC-I molecules and retain a functional antigen presentation machinery (Silvestre et al., 1970; Goh et al., 2007; Wilson et al., 2016). This led to Junqueira et al. discovering that *P. vivax*-infected reticulocytes express MHC-I which are recognized by CD8+ T cells, resulting in the cytotoxic killing of infected reticulocytes (Junqueira et al., 2018). Similar observations were made with *P. yoelii*-infected reticulocytes in mice (Jayawardena et al., 1983; Hojo-Souza et al., 2020). It is worth mentioning that CD8+ T cells have also been implicated to play a role in the clearance of infected murine erythroblasts (Imai et al., 2013, 2015).

**Figure 1:**
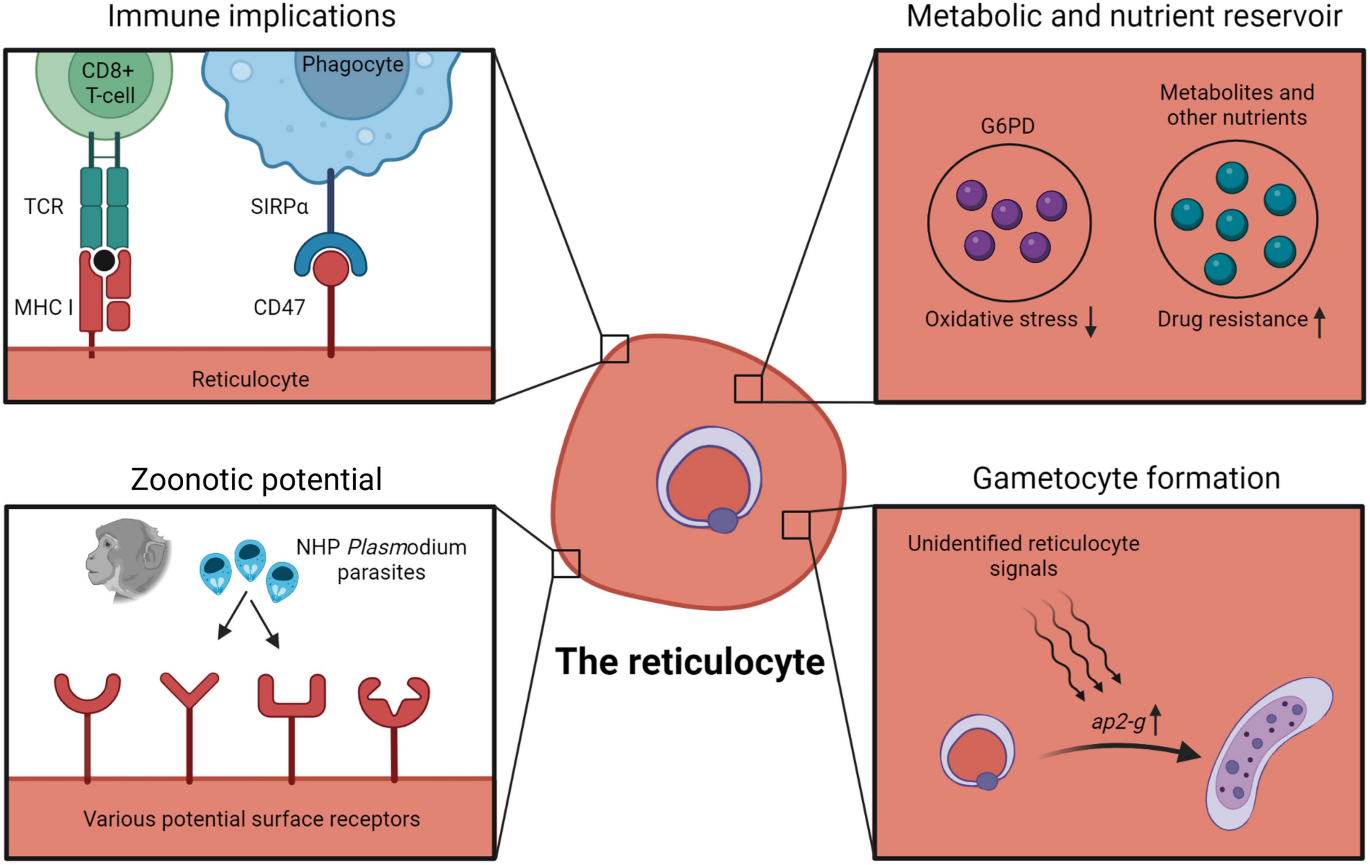
Advantages, disadvantages and implications of reticulocyte tropism. Infected reticulocytes have been shown to be recognizable by host CD8+ T cells due to MHC-I expression of reticulocytes. Early reticulocytes also express CD47 as a “do not eat me” signal to host phagocytes. Higher intracellular G6PD levels in reticulocytes has been postulated to protect parasites against oxidative damage. The relatively nutrient-rich microenvironment of the reticulocyte could also be capitalized by the parasite, and in some cases, enables the parasite to bypass metabolic pathways targeted by antimalarial drugs. Besides, observations with *P. knowlesi* and *P. cynomolgi* suggest that human reticulocytes might act as “gateways” to zoonotic transmissions due to the wide variety of potential receptors on reticulocyte surfaces. Data showing increased gametocyte formation in reticulocytes also hint at unidentified reticulocyte-specific signals inducing gametocyte conversion. Figure was created in Biorender.com.

In contrast to the increased susceptibility to CD8+ T cells, reticulocytes also express higher levels of CD47, the ‘do not eat me’ signal, and parasitizing CD47-high reticulocytes was suggested as an immune evasion strategy to escape phagocyte clearance (Banerjee et al., 2015). Mice knocked-out for CD47, or treated with CD47-neutralizing antibodies, harbored lower parasite burden, in part due to the increased phagocytosis of infected erythrocytes by splenic macrophages. From these studies, it is not immediately clear whether parasitizing reticulocytes is an effective immune evasion strategy, due to the complex interactions of the infected reticulocytes with multiple different aspects of the host immune system (Figure 1).

### Gametocyte formation

Fascinatingly, reticulocyte tropism could also contribute to gametocyte formation. *P. falciparum* gametocyte formation *in vitro* was 10 times higher in reticulocyte-rich blood compared to control blood (Trager et al., 1999). Phenylhydrazine-induced reticulocytosis also resulted in higher gametocyte formation in mice infected with *P. berghei* and *P. chabaudi*, but not *P. yoelii* and *P. vinckei* (Gautret et al., 1996, 1997). However, these older studies did not show that the increased gametocytogenesis occurred specifically in reticulocytes instead of normocytes.

To address this, by comparing the transcriptome of reticulocyte-resident and normocyte-resident parasites using single-cell RNA sequencing, Hentzschel et al. detected the upregulation of gametocyte-related genes in reticulocyte-infecting parasites (Hentzschel et al., 2022), with the most notable being *ap2-g*, the master regulator of *Plasmodium* sexual commitment (Kafsack et al., 2014; Sinha et al., 2014; Poran et al., 2017). These transcriptomic data were verified experimentally, in which freshly invaded erythrocytes were sorted into CD71+ reticulocyte and CD71- normocyte fractions, and they found that the gametocyte conversion rate in the CD71+ reticulocyte fraction was significantly higher. It seems likely that an unknown reticulocyte factor acts as a trigger for sexual commitment through the regulation of *ap2-g* (Figure 1).

Balancing between sexual commitment and asexual replication is critical for the parasite to ensure efficient transmission while maintaining persistent infection in the vertebrate host. Could reticulocyte tropism be a strategy to regulate this balance? Invasion of the scarcely available reticulocytes could ensure that only a small proportion of the parasite population is predisposed to sexual commitment. Of course, the data so far show that parasites within normocytes can also develop into gametocytes, implying that erythrocyte tropism is just one of many factors affecting sexual commitment. Regardless, sexual commitment within reticulocytes makes survival sense, at least for *P. falciparum*; the younger and more metabolically-rich reticulocyte could better support the longer gametocyte developmental time of 9–12 days, as compared to the 48 h asexual cycle (Hawking et al., 1971). *P. vivax* gametocytes, however, mature much faster than *P. falciparum* gametocytes, only taking 2–4 days (McCarthy et al., 2013). Could the reticulocyte microenvironment contribute to this accelerated gametocyte maturation? To address this, a direct comparison of gametocyte development between *P. falciparum*-infected reticulocytes and *P. falciparum*-infected normocytes is necessary.

Although here we focus on reticulocyte and normocyte tropism, it is worth mentioning that late erythroblasts might also be an important host cell for gametocyte development. Neveu et al. observed that *P. falciparum* gametocytes can fully develop within late erythroblasts (polychromatic and orthochromatic stages; Neveu et al., 2020). Even more surprisingly, they found that parasites delayed erythroblast maturation, resulting in mature infective gametocytes within reticulocytes, and the timing coincides with the release of the infected reticulocyte from the bone marrow. It is tempting to speculate that this allows gametocytes to develop safely in the hematopoietic niches, away from threats of the immune system and splenic filtration.

### Metabolic reservoir and drug resistance

The reticulocyte is more metabolically complex than normocyte. Besides higher levels of various amino acids, nucleotides, sugars and lipids, reticulocytes also retain some active metabolic pathways (Malleret et al., 2013; Srivastava et al., 2015, 2017). This relatively rich microenvironment could be capitalized by the parasite in multiple ways. *P. berghei* mutant parasites with compromised pyrimidine biosynthesis could only grow in young reticulocytes, presumably being able to scavenge pyrimidines from the host reticulocytes to support its growth (Srivastava et al., 2015). Similarly, although hemoglobin digestion is vital for parasite development, Lin et al. unexpectedly managed to generate *P. berghei* mutant lines with impaired hemoglobin digestion, and these mutants were restricted to growth within reticulocytes (Lin et al., 2015). It is unclear how these mutants were able to develop within reticulocytes without hemoglobin as a source of amino acids, but possible explanations include scavenging on the reticulocytes’ amino acid reservoir or importing amino acids from the extracellular environment. More importantly, as the antimalarial drug chloroquine works by disrupting hemozoin formation resulting in the toxic buildup of heme (byproduct of hemoglobin digestion), these mutants were chloroquine-resistant due to minimal hemoglobin degradation. A separate study also linked chloroquine resistance with reticulocyte tropism; the authors suggested that the higher glutathione levels in reticulocytes were able to detoxify heme, precluding the need for hemozoin formation and resulting in the chloroquine resistance of reticulocyte-residing parasites (Platel et al., 1999). Altogether, these findings indicate that reticulocyte tropism could result in resistance against antimalarial drugs that target essential parasite metabolic pathways, by relying on the reticulocyte metabolic reservoir (Figure 1).

### G6PD and oxidative stress

Glucose-6-phosphate dehydrogenase (G6PD) is an enzyme in the pentose phosphate pathway. Besides being critical for energy production in erythrocytes, G6PD also protects the cells from oxidative damage. Reticulocytes contain about five times more G6PD than normocytes (Wilson et al., 2016). As suggested by some, this higher G6PD activity in reticulocytes could provide malarial parasite better protection from oxidative damage (Gunalan et al., 2020; Neveu and Lavazec, 2021; Figure 1). This is supported by an *ex vivo* study with blood from G6PD-deficient patients; even though their normocytes lacked G6PD activity, their reticulocytes showed similar G6PD activity to wildtype erythrocytes (Bancone et al., 2017b). Reticulocytes from G6PD-deficient patients were also able to support normal *P. vivax* invasion, late-stage development and gametocyte formation. It is worth noting, however, that a separate longitudinal and association study found a strong correlation between G6PD deficiency and lower *P. vivax* parasite density (Louicharoen et al., 2009). Needless to say, external factors beyond erythrocyte G6PD levels could have contributed to the lower parasite burden. Nonetheless, there is an urgent need to assess the impact of G6PD deficiency on malaria, especially considering that G6PD deficiency is the most widespread enzymatic disorder with many overlapping regions with malaria distribution. The fact that reticulocytes of G6PD-deficient patients have normal G6PD activity means that reticulocyte levels should be accounted for in studies investigating the link between G6PD deficiency and malaria (Bancone et al., 2017a).

### Zoonotic potential

Earlier we discussed the erythrocyte tropisms of the zoonotic *P. knowlesi* and *P. cynomolgi*. Both showed no particular inclination for macaque erythrocyte types, however, they displayed a strong preference for human reticulocytes over normocytes (Lim et al., 2013; Amir et al., 2016; Kosaisavee et al., 2017). These observations suggest that human reticulocytes can act as ‘gateways’ for NHP malaria to be zoonotically transmitted to humans. The relatively larger surface proteome of reticulocytes could mean that it is likelier for NHP-infecting merozoites to bind and enter human reticulocytes (Figure 1). The more metabolically rich microenvironment of the reticulocyte could also enable the NHP parasites to adapt better within a human host erythrocyte. Of particular note, when *P. knowlesi* was adapted *in vitro* to grow in human erythrocytes (mostly normocytes) over a long period, the parasites were able to invade human normocytes as well as reticulocytes (Lim et al., 2013). The plasticity of zoonotic malaria species tropism is a cause for concern and should encourage studies aiming to understand how NHP malaria strains invade human reticulocytes (Hang et al., 2021).

### Membrane stability

Reticulocyte maturation involves extensive membrane remodeling, a process that could be hijacked by the parasites to remodel reticulocytes for their own means, possibly conferring an advantage to invading reticulocytes. During parasite development, the parasite remodels the host erythrocyte membrane by establishing new permeability pathways (NPPs) to acquire essential nutrients from the extracellular environment. NPPs increase the permeability of the erythrocyte membrane to certain solutes, but also compromise membrane integrity resulting in lower osmotic stability (Ginsburg et al., 1983; Lew et al., 2004; Clark et al., 2021). Additionally, reticulocytes are more osmotically stable than normocytes (Bernecker et al., 2019; Clark et al., 2021). In fact, one reticulocyte enrichment method involves a short exposure to hypotonic saline to lyse normocytes while the reticulocytes remained intact (Grimberg et al., 2012). This additional stability could be advantageous to reticulocyte-infecting parasites, especially during late into intraerythrocytic development when the infected cells are most prone to lysis due to NPPs. In other words, reticulocyte stability could mean that the cells are more resilient and accommodative of the parasite’s extreme remodeling, without compromising cell survival. On the contrary, Clark et al. estimated that *P. vivax*-infected reticulocytes were about 70% less stable to osmotic lysis compared to *P. falciparum*-infected normocytes (Clark et al., 2021). However, species-specific factors could have played a role in their observations, and a fairer comparison would have been between *P. falciparum*-infected reticulocytes and *P. falciparum*-infected normocytes, to really delineate the relative contributions of the host cell type to osmotic stability.

Membrane remodeling of the infected cell also impacts membrane deformability. Remarkably, the deformability of *P. vivax*-infected reticulocytes and *P. falciparum*-infected normocytes are affected differently: *P. vivax* increases reticulocyte deformability (Suwanarusk et al., 2004; Handayani et al., 2009; Malleret et al., 2015), whereas *P. falciparum* decreases normocyte deformability (Cranston et al., 1984; Glenister et al., 2002; Suwanarusk et al., 2004). The decreased deformability of late-stage *P. falciparum*-infected cells predisposes them to splenic clearance but this is circumvented by sequestering in microvasculatures (Smith et al., 2013; Lee et al., 2019). For *P. vivax*, the increased deformability of infected reticulocytes could abolish the need for late-stage parasite sequestration, as supported by *ex vivo* data showing that *P. vivax*-infected cells cytoadhered to endothelial cells 10 times less often than *P. falciparum*-infected cells (Carvalho et al., 2010). Again, it is unclear whether this difference in deformability is due to host cell type or parasite-specific modifications, and a side-by-side comparison between *P. falciparum*-infected reticulocytes and *P. falciparum*-infected normocytes should clarify this.

## Future directions of tropism research

From our comprehensive review of past erythrocyte tropism studies, it is clear that reticulocyte tropism is more frequent than once thought (if not the norm) among *Plasmodium* spp. Further accentuating this is our increasing realization of the importance of reticulocyte-rich hematopoietic niches as parasite repositories. However, not much is known regarding how merozoites sense and differentiate reticulocytes from normocytes, whether it is by receptor recognition or through unknown mechanisms (Box 2). This is especially apparent from *P. falciparum*’s preference for human reticulocytes; although many receptors for *P. falciparum* have been identified, none explains its reticulocyte tropism. Unfortunately, this also means that interventions targeting *P. falciparum*’s erythrocyte invasion, which are based on current known receptor-ligand interactions, might be ineffective at preventing reticulocyte invasion. Similarly, antimalarial drugs which work well with normocyte-resident parasites might not be able to target reticulocyte-resident parasites due to the presence of alternative metabolic pathways in the reticulocyte nutrient-rich microenvironment. This could also be one of the reasons why during the drug discovery process, many compounds with good antimalarial activity shown by *in vitro* screens (using normocyte-adapted *P. falciparum* lab strains) fail to show the same efficacy in rodent models (commonly with the reticulocyte-preferring *P. berghei*; Fidock et al., 2004). Undoubtedly, the reticulocyte’s role as a potential intervention escape mechanism must be considered at some point during drug discovery and vaccine design. In fact, interventions which directly target reticulocyte invasion could be a whole new strategy in malaria control and elimination.

BOX 2. **Outstanding questions on *Plasmodium* erythrocyte tropism.**

- How do malarial parasites sense and invade reticulocytes?
- How should the process of drug discovery and vaccine design adapt to account for reticulocyte tropism?
- Are infected reticulocytes more resistant to clearance by the host immune system and spleen?
- How do parasites capitalize on the reticulocyte microenvironment?
- What is the link between reticulocyte tropism and hematopoietic niche tropism?

Although we have discussed some possibilities in this review, it is still unclear whether reticulocyte tropism provides a survival advantage to malarial parasites (Box 2). Are infected reticulocytes more resistant to the host immune system and splenic clearance? Do reticulocytes provide a more accommodating environment for the intracellular development of the parasite? Answering these questions, however, is extremely challenging considering the diverse cellular differences between reticulocytes and normocytes. Dissecting how individual aspects of the reticulocyte contribute to parasite survival would require a constant homogenous source of reticulocytes (possibly circumvented by using *in vitro*-generated reticulocytes; Trakarnsanga et al., 2017; Lim et al., 2021; Sivalingam et al., 2021; Yu et al., 2022) and methods to experimentally manipulate relevant reticulocyte cellular properties. Regardless, addressing these critical questions is the way forward.

## Author contributions

YL and LR conceptualized the review. YL, BR, BM, and LR wrote the draft. All authors contributed to the article and approved the submitted version.

## Funding

LR is funded by the Singapore National Medical Research Council IRG Grant (NMRC/OFIRG/0065/2018), core grant given to A*STAR ID Labs by A*STAR, and by the Singapore Ministry of Education (Start-up University grant #022388-0001).

## Conflict of interest

The authors declare that the research was conducted in the absence of any commercial or financial relationships that could be construed as a potential conflict of interest.

## Publisher’s note

All claims expressed in this article are solely those of the authors and do not necessarily represent those of their affiliated organizations, or those of the publisher, the editors and the reviewers. Any product that may be evaluated in this article, or claim that may be made by its manufacturer, is not guaranteed or endorsed by the publisher.
